# Encapsulating IM7-Displaying Yeast Cells in Calcium Alginate Beads for One-Step Protein Purification and Multienzyme Biocatalysis

**DOI:** 10.3389/fbioe.2022.849542

**Published:** 2022-03-17

**Authors:** Wenhao Yin, Xinping Wang, Ying Liao, Lixin Ma, Jie Qiao, Hui Liu, Xin Song, Yi Liu

**Affiliations:** ^1^ School of Life Science and Technology, Wuhan Polytechnic University, Wuhan, China; ^2^ State Key Laboratory of Esophageal Cancer Prevention and Treatment, Zhengzhou University, Henan, China; ^3^ State Key Laboratory of Biocatalysis and Enzyme Engineering, School of Life Sciences, Hubei University, Wuhan, China; ^4^ Department of Hematology, Renmin Hospital of Wuhan University, Hubei, China; ^5^ BravoVax Co., Ltd., Hubei, China

**Keywords:** Colicin E7/IM7 affinity chromatography, one-step protein purification, multiple-enzyme complex, biocatalysis, enzyme immobilization

## Abstract

There are several commercial chromatographic systems for protein purification; however, development of cost-effective 3H-grade (high yield, high purity, and high activity) purification approaches is highly demanded. Here, we establish a methodology for encapsulating the IM7-displaying yeast cells in calcium alginate beads. Taking advantage of this biomaterial-based affinity chromatography, rapid and cost-effective purification of proteins with over 90% purity in a single step is achieved. Moreover, our system enables coating the multienzyme complex to produce reusable immobilized cells for efficient cascade biotransformation. Together, the present method has great application potentials not only in the laboratory but also in the industry for production of protein products as well as biocatalysis.

## Introduction

For most biological studies, obtaining protein samples with high yield, high purity, and high activity (3H purification) is the first and most critical step. On the one hand, there are plenty of commercial chromatography systems (Fong et al., 2010; Kimple et al., 2013) for purifying proteins, yet many of them have low capacity and command a high price, which barely meet the requirement of high yield necessary for commercial protein production. In addition, most of the chromatography systems (Lichty et al., 2005) adopt chemical matrix (e.g., Sepharose or silica) as the packing material, resulting in the requirement of frequent replacement. On the other hand, it is still difficult to obtain high-quality protein products in a single step by using commercial columns. An average purification protocol typically includes ∼3 chromatographic steps (Lichty et al., 2005), causing more than 50% protein loss during multiple purification steps. Therefore, development of convenient and cost-effective 3H-grade purification methods is highly demanded. In Table 1, we include and compare the common protein purification methods.

**TABLE 1:** Comparison of the commonly used chromatographic systems.

Type	Affinity *K*_d_ (M)	Loading NaCl (M)	Capacity (mg/ml)
FLAG	10^–8/−9^	<0.2	0.6
Strep	10^–6^	<0.2	6
MBP	10^–6^	<0.2	6–10
GST	10^–6/−7^	<0.2	10
His	10^–8/−9^	1+	20–40
CL7 (this work)	10^–14/−17^	1+	10–15

Note: FLAG, antibody-binding peptide; Strep, streptavidin-binding peptide; MBP, maltose-binding protein; CL7, engineered CE7 tag.

Recently, we established an indirect *Pichia pastoris* surface display method (Li et al., 2019; Dong et al., 2020) based on the ultra-high–affinity complex between the engineered Colicin E7 DNase (CL7) and its inhibitor, immunity protein 7 (IM7) (Ko et al., 1999). The IM7 proteins, which can tightly bind to recombinant proteins with CL7 fusion tags, were anchored three times on the surface of yeast cells, achieving high-efficiency–displaying enzymes or a multienzyme complex for catalysis (Dong et al., 2020). Additionally, Vassylyeva et al. developed a chromatographic technology for efficient purification of complex proteins by using the IM7/CL7 protein pair (Vassylyeva et al., 2017). Compared to the existing affinity protein pairs, the IM7/CL7 system has many advantages. For instance, the affinity between IM7 and CL7 (*K*_d_∼10^–14^–10^–17^ M) is approaching that of a covalent bond, which is 4–7 orders of magnitude higher than that of any other available analogs (Table 1), including the most widely used His-tag (*K*_d_∼10^−8^–10^–9^ M). Besides, the most protein ligation methods only utilize small peptides as the tags. For example, the SpyCatcher–SpyTag system (Hatlem et al., 2019) employs a 13-amino-acid peptide (SpyTag), which cannot enhance the protein yield and solubility as the CL7 tag does. Moreover, we recently found that fusion of CL7 tag can also significantly improve the stability of the target proteins.

To achieve efficient biocatalysis, natural biosynthetic pathways are often designed to couple and balance sequential reactions by increasing local enzyme or substrate concentrations, or by limiting diffusion of unstable or toxic intermediates. Hence, rational design of multienzyme complex for cascade biotransformation is of great importance. A classic case is to construct artificial cellulosomes within microorganisms (e.g., yeast) for conversion of cellulose into fermentable sugars. We have harnessed the IM7/CL7 protein pair to engineer *Pichia pastoris* with minicellulosomes (Dong et al., 2020), enabling direct conversion of CMC to bioethanol. To sum up, in this work, we aim to develop a biomaterial-based and cost-effective method for protein purification as well as for construction of a multiple-enzyme complex for biocatalysis.

Firstly, we looked through the literature studies about the existing encapsulation techniques for preservation applications (Cassidy et al., 1996; Subramani and Ganapathyswamy, 2020). Among these techniques, immobilization of microbial cells by entrapment in calcium alginate (Ca-alginate) gels is the most widely used one and is attractive for a variety of applications in biotechnology, biomedicine, and food technology (Kregiel et al., 2013; Rokstad et al., 2014). So, we propose calcium alginate as chassis to fabricate porous beads. Specifically, we report a method which can efficiently encapsulate the IM7-displaying yeast cells by calcium alginate, producing porous beads as the packing material for chromatography columns. Employing these yeast-encapsulating beads, we achieved rapid purification of the high-purity proteins in a single step. Moreover, we harnessed the method to coat the multienzyme complex for high-efficiency biocatalysis. Taken together, the presented methodology has great potential for large-scale production of biologically active proteins by the pharmaceutical industry as well as for construction of cell factory for biocatalysis.

## Materials and Methods

### Materials

Fluorescein isothiocyanate (FITC) was purchased from Solorbio (Shanghai, China). Lipofectamine CRISPMAX was purchased from Thermo Fisher (China). The serum mediums were purchased from Natocor (Wuhan, China). All the other chemical reagents were purchased from Sigma-Aldrich (Mainland, China). *E. coli* DH5α was used as the host for DNA manipulations, while *E. coli* BL21 (DE3) was the host for recombinant expression of CL7-tagged eGFP, Cas9, cellulases, and CBM domains. The *P. pastoris* strain GS115 and the vector pPICZαA were obtained from Invitrogen (Carlsbad, CA, United States). The vectors pET23a-T and pET23a-CL7 were constructed and stored in our laboratory previously (Li et al., 2019; Dong et al., 2020). In addition, the plasmids to express CL7-tagged eGFP and Cas9 (Qiao et al., 2019) were constructed before. The genes encoding exo-mode cellobiohydrolases (CBH) from *Yarrowia lipolytica*, endoglucanase (EG) from *Clostridium thermocellum* DSM1237, glucose-tolerant β-glucosidase (BGL) from *Thermoanaerobacterium thermosaccharolyticum* DSM 571, and CBM from *Thermobifida fusca* were synthesized by Sangon Biotech (Shanghai, China). The plasmids of the above-mentioned cellulases and CBM were all constructed before (Dong et al., 2020).

The *E. coli* strains were grown in LB medium (1% tryptone, 0.5% yeast extract, 1% NaCl) supplied with 100 μg/m ampicillin. *P. pastoris* yeasts were grown firstly in YPD plates (1% yeast extract, 2% peptide, and 2% glucose) supplemented with 100 µg/ml of Zeocin and then cultured in BMGY/BMMY medium base (20.0 g/L peptone, 10.0 g/L yeast extract, 100 mmol PBS broth, pH 6.0).

### Yeast Surface Display and *E. coli* Expression

The plasmids of IM7-displaying yeast were digested with Pme1 and transformed into GS115 competent cells. Transformants were firstly isolated by incubation at 28°C for 48 h on YPD plates supplemented with 100 µg/ml of Zeocin. Then, five to ten single colonies of transformants were inoculated in 20 ml of BMGY in 250 ml flasks and cultivated at 28°C under 200 rpm. After 24 h, the cells were centrifuged at 5,000 × g for 5 min, resuspended in 20 ml of BMMY medium containing 1% (v/v) methanol, and continued to grow at 28°C and 200 rpm for 24 h.

To express the CL7-tagged proteins in *E. coli*, 0.5 mM isopropyl-β-D-thiogalactopyranoside (IPTG) was added to the cells when the cells were grown to an OD_600_ of 0.6. Then, the cells were grown at 18°C for 12 h. The *E. coli* cells collected were resuspended in PBS buffer containing 200 mm NaCl and 10 mm CaCl_2_ (pH 7.4) and then sonicated on ice for 20 min. The cell lysates were either purified by Ni-NTA affinity columns or directly incubated with the Ca-alginate–encapsulated yeast strains.

### Fluorescence Microscopy and Flow Cytometric Analysis

The yeast strains were harvested and washed twice by ice-cold water, resuspended, and blocked in 1 ml PBS buffer (200 mM NaCl, pH 7.4) with 1 mg/ml BSA for 1 h at 4°C with rotation. Then, 1 µL of mouse anti-HA tag monoclonal antibodies was added to the cell suspension of 1,000 µL and then incubated at room temperature with rotation for 2 h. In the next step, the cells were washed three times with PBS and resuspended in 200 µL of PBS with the addition of 1 µL of FITC-conjugated goat anti-mouse IgG (H + L) antibodies, followed by incubation of them at room temperature for 1 h with rotation. Finally, the cells were washed three times with PBS, resuspended in 1 ml of PBS, and examined by fluorescence microscopy (IX73, Olympus, Tokyo, Japan). Flow cytometric analysis (FACS) was analyzed with a flow cytometer (CytoFLEX, Beckman Coulter, Suzhou, China) to estimate the percentage of the fluorescence-positive IM7-displaying yeast cells.

### Encapsulating IM7-Displaying Yeast Cells in Ca-Alginate Beads

Ca-alginate yeast-encapsulated beads were prepared by dropping a mixture of sodium alginate solution and yeast cells (10 g/L) into a saturated boric acid containing 4% (w/w) CaCl_2_ solution under slow magnetic stirring at 25°C. The resulting beads were 3 ± 0.2 mm in diameter and stirred in this solution for 30 min. They were then collected by filtration, washed three times with sterile and deionized water, and stored in Tris-HCl buffer (pH 7.0) at 4°C.

### Scanning Electron Microscopy

Samples of SA@yeast beads were coated under vacuum with a thin layer of gold and examined by scanning electron microscopy (SEM) (JSM 6510LV, JEOL, Japan) at 10–20 kV. Electron microscopy showed that yeast cells were attached to the Ca-alginate beads in the inner region.

### *In Vitro* Plasmid DNA Cleavage Assay

The target plasmid DNA was synthesized and inserted into a pET23a(+) vector. For a typical cleavage assay, the Cas9 RNP (300 ng) was diluted into the cleavage buffer (25 mM Tris-HCl, pH 8.0, 150 mM KCl, 5 mM MgCl_2_, 5 mM DTT, 5% glycerol). The plasmids (200 ng) were cleaved in the cleavage buffer. The mixture was incubated at 37°C for 30 min, and then the temperature was elevated to 85°C for 5 min to stop the reaction. The cleavage products were analyzed by agarose gels and viewed by GoldView staining.

### Enzyme Activity Assay

The enzyme activity of cellulosome or free cellulases against microcrystalline cellulose (Avicel) or phosphoric acid–swollen cellulose (PASC) or carboxymethyl cellulose (CMC) was detected by 3,5-dinitrosalicylic acid (DNS) assay (Bailey et al., 1992). The PASC (86.2) was prepared from Avicel (Sangon Biotech, Shanghai, China) as described previously (Zhang et al., 2006). Minicellulosomes or free cellulases were incubated with 0.1% cellulose substrate in 50 mM citrate buffer (pH 4.8) with 10 mM CaCl_2_ at 50°C for 30 min. After addition of DNS and boiling for 10 min, the reducing sugars were detected at 540 nm. One unit of the enzyme activity was defined as the amount of enzyme that released 1 micromol of product from the cellulose substrate at 50°C in 1 min.

## Results and Discussion

### Preparation of Ca-Alginate Yeast-Encapsulated Beads

**FIGURE 1:**
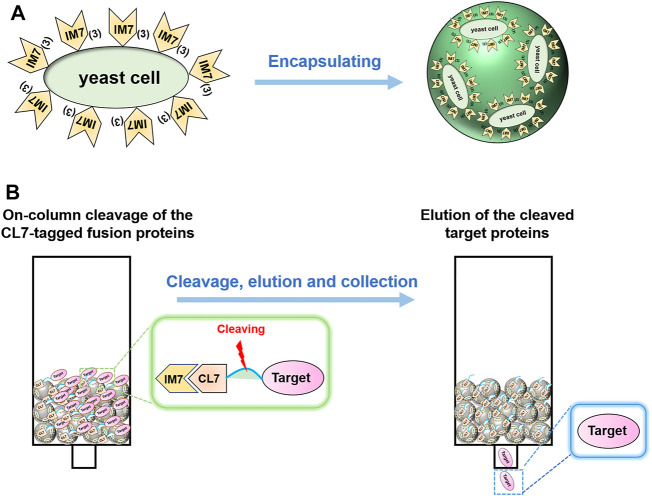
**(A)** Schematics of encapsulating the IM7-displaying yeast cells by calcium alginate. **(B)** Flow diagram of one-step protein purification by using the Ca-alginate yeast-encapsulated beads.

**FIGURE 2:**
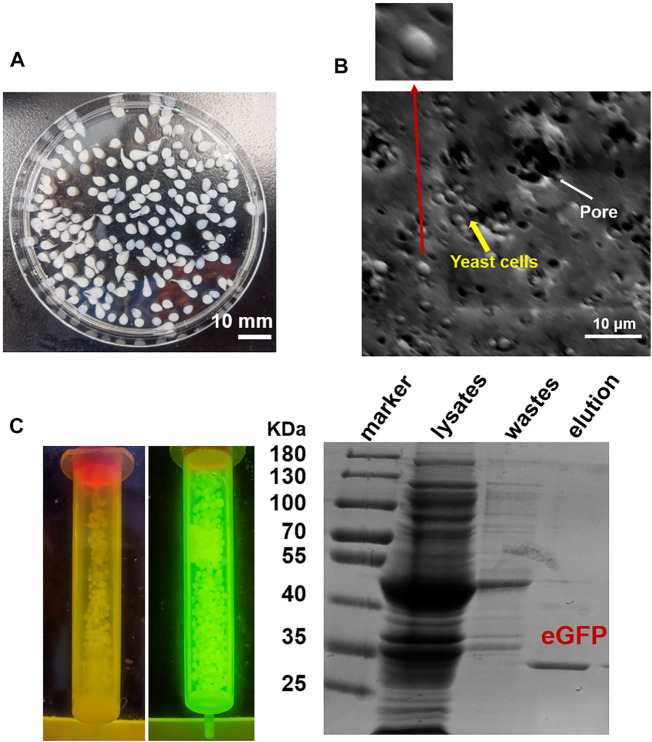
**(A)** Image of SA@yeast beads. **(B)** Scanning electron microscopy (SEM) image of SA@yeast beads after air-blast drying. The inset shows a magnification of one yeast cell. **(C)** Purification of eGFP by using SA@yeast beads. (Left) Blue light transmitter analysis of SA@yeast beads without or with CL7-eGFP. (Right) 10% SDS-PAGE analysis of *E. coli* cell lysates, washing wastes, and eluted eGFP.

Firstly, we tested the possibility to directly adopt the IM7-displaying yeast cells (Supplementary Figure S1) to pack chromatograph columns for purification of the target proteins. Unfortunately, the elution efficiency of protein is very low in this way since the yeast cells are possibly packed too tightly to block the column. To solve the issue, we decided to encapsulate the engineered IM7-displaying yeast cells in calcium alginate beads (Figure 1A) and then use them for protein purification (Figure 1B). After many assays (Supplementary Table S1), we found that the best coating solution is mixing the IM7-displaying yeast cells with the solutions containing 2.5% (w/w) sodium alginate (SA) (Fan et al., 2017; Kim et al., 2017; Benucci et al., 2019), 0.5% (w/w) gelatin, and 2% (w/w) polyvinyl alcohol (PVA). Meanwhile, the saturated boric acid containing 4% CaCl_2_ was employed as the cross-linking reagent. As a result, the Ca-alginate yeast-encapsulated beads (hereafter named “SA@yeast beads”) were constructed with an average diameter of 3 mm (Figure 2A). The SEM image (Figure 2B) shows the interiors of these beads, clearly indicating that the yeast cells were well encapsulated. Importantly, there are plenty of pores inside the beads, allowing the free proteins to move in and out efficiently. This is the foundation for our method.

### One-Step Protein Purification by Using the SA@yeast Beads

**FIGURE 3:**
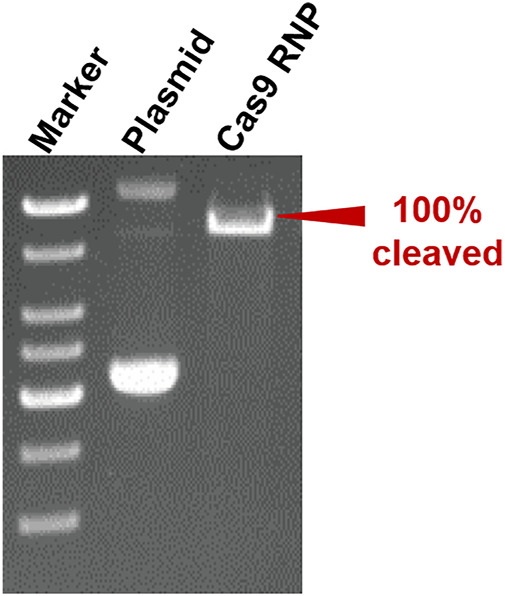
Result of cleavage of the target plasmid by using Cas9 RNPs (0.8% agarose gel).

To verify the versatility of our method, we harnessed the SA@yeast beads to purify various proteins with different sizes, including eGFP (enhanced GFP) (∼27 kDa) (Zhang et al., 2017), *Streptococcus pyogenes* Cas9 (spCas9, ∼170 kDa) (Jinek et al., 2012), and *Yarrowia lipolytica* exo-mode cellobiohydrolase (Wei et al., 2014) (CBH, ∼100 KDa). Following a standard procedure, the *Escherichia coli* cell lysates were incubated with the beads and washed by a high-salt buffer containing 500 mM NaCl. To remove the N-terminal CL7 tag of the target protein, an engineered human HRV 3C proteinase with a CL7 tag was added for on-column cleavage followed by collecting the elution. As shown in Figure 2C, the eGFP proteins were purified in a single step with a purity of over 95%. Similar results were observed for both Cas9 and CBH (Supplementary Figure S2), demonstrating that our method can be used as a universal tool for protein purification. The protein loading capacity of this CL7/IM7 yeast affinity system is ∼10–15 mg per 1 ml SA@yeast beads (Table 1). Compared with the Ni-NTA affinity purification approach, the CL7/IM7 affinity system significantly increases the purification level of the end protein products (Supplementary Figure S3). More importantly, it maintains or improves the activity of the protein products (Vassylyeva et al., 2017). For instance, the purified Cas9 enzymes made by this method show excellent endonuclease activity (Figure 3).

### Coating Multienzyme Complex for Efficient Biocatalysis

**FIGURE 4:**
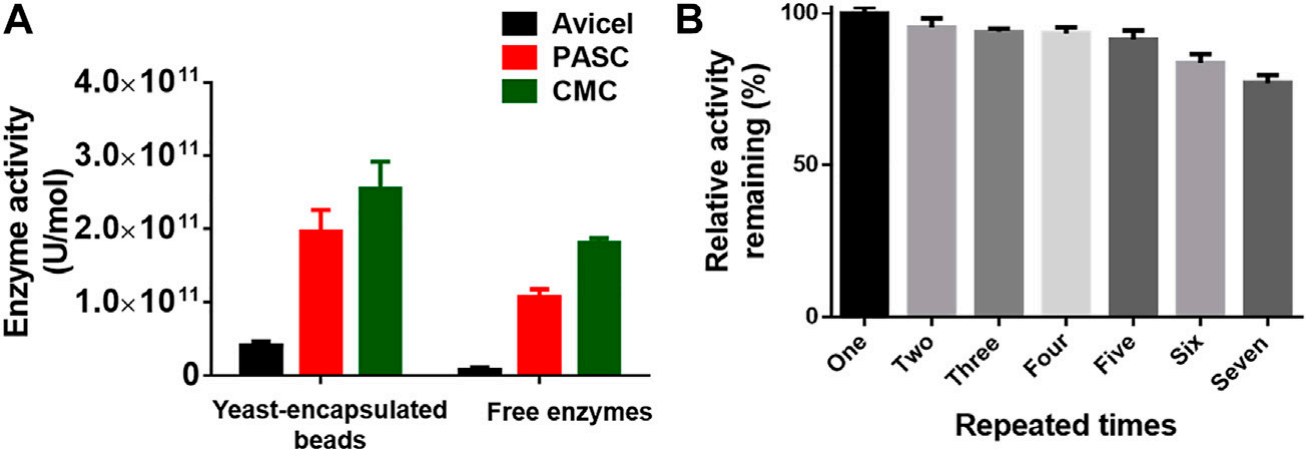
**(A)** Enzyme activity of the encapsulated yeast cells and free enzymes against different cellulose substrates. **(B)** Remaining enzyme activity of the encapsulated yeast cells after repeated use. The duration for each assay is ∼40 min.

To extend the scope of the method, we adopted the system to coat the multienzyme complex for biocatalysis. The cellulosome, a complicated multienzyme machine produced by cellulolytic microorganisms, was chosen to perform the proof-of-concept experiment. To achieve efficient carboxymethyl cellulose hydrolysis, three cellulases including an endoglucanase (EG) from *Clostridium thermocellum* DSM1237 (Leis et al., 2017), a glucose-tolerant β-glucosidase (BGL) from *Thermoanaerobacterium thermosaccharolyticum* DSM 571 (Zhao et al., 2013), and a CBH from *Yarrowia lipolytica* (Wei et al., 2014), as well as a carbohydrate-binding module (CBM) from *Thermobifida fusca* (Forsberg et al., 2014), were fused with an N-terminal CL7 tag and over-expressed in *E. coli*. Then, the IM7-displaying yeast cells were *in vitro* incubated with the *E. coli* lysates containing three cellulases and CBM at a ratio of 1:1:1:1 (Dong et al., 2020), leading to assembly of minicellulosomes on the surface of these yeast cells. Next, those yeast cells were encapsulated in Ca-alginate beads, and their cellulase activity against Avicel, phosphoric acid–swollen cellulose [PASC (86.2)], and carboxymethyl cellulose (CMC) was determined by measuring the reducing sugars within the first 30 min. Compared with free cellulases in solution, the coated multienzyme complex exhibited significantly enhanced catalytic activity (Figure 4A). More importantly, these SA@yeast beads can be repeatedly used for at least six times with very slight activity decreases (Figure 4B), demonstrating great potential in the production of cellulosic ethanol in the future.

## Conclusion

Currently, there are plenty of commercial chromatography columns for protein purification in the market. However, most of them are costly and cannot be prepared in the laboratory. To resolve this issue, here we developed a method to simply perform biomaterial-based affinity chromatography. Specifically, the IM7-displaying yeast cells were encapsulated in the porous Ca-alginate beads. Taking advantage of the super-affinity IM7/CL7 protein pair, one-step purification of protein products with ∼95% purity was achieved. More importantly, the approach benefited the purification of low-expression proteins. Compared to immobilizing purified IM7 proteins, direct encapsulation of yeast cells is much more convenient and cost-effective.

We also coated the yeast cells with the surface-displaying multienzyme complex for biocatalysts. As an example, the yeast cells with artificial minicellulosomes were encapsulated in the Ca-alginate beads. Compared with free cellulases, the SA@yeast beads showed significantly improved catalytic activity against cellulose substrates. Moreover, the SA@yeast beads can be repeatedly used several times. In the future, we may adopt these yeast-encapsulating biomaterials for whole-cell catalysis to produce valuable compounds.

In conclusion, we developed a methodology for encapsulating the IM7-displaying yeast cells in Ca-alginate beads. The system can be applied to 3H-grade purification of protein products and reusable biocatalysis, showing great potential in the field of pharmaceutical and bioengineering industry.

## Data Availability

The raw data supporting the conclusions of this article will be made available by the authors, without undue reservation.
