# Supplementary material for: Encapsulating IM7-Displaying Yeast Cells in Calcium Alginate Beads for One-Step Protein Purification and Multienzyme Biocatalysis
Source: Front Bioeng Biotechnol. 2022 Mar 17;10:849542. doi: 10.3389/fbioe.2022.849542 (PMC8969745; doi:10.3389/fbioe.2022.849542)
Supplement: Supplementary file 1 [file DataSheet1.docx]

**Table S1.** Effects of embedded materials on the properties of microspheres.

| NO. | Sodium alginate % | Geltatin % | PVA % | Diameter (mm) | Density^a^ (g. cm^3^) | Relative Mass transfer perfomance^d^ |
| --- | --- | --- | --- | --- | --- | --- |
| 1 | 0.5 | 0.5 | 2 | N/A^b^ | N/D^c^ | N/D |
| 2 | 0.5 | 1 | 4 | N/A | N/D | N/D |
| 3 | 1.5 | 0.5 | 2 | trailing | N/D | N/D |
| 4 | 1.5 | 1 | 4 | 2.8 | 0.92 | 1 |
| 5 | 2.5 | 0.5 | 2 | 3.0 | 0.87 | 4 |
| 6 | 2.5 | 1 | 4 | 3.2 | 0.89 | 2 |
| 7 | 3.0 | 0.5 | 2 | 3.8 | 0.94 | 2 |
| 8 | 3.0 | 1 | 4 | 3.9 | 0.97 | 3 |

1. Weigh a cylinder containing a certain volume of water, record its initial mass, and then put 40 SA@yeast beads with similar size in this cylinder. The final mass and volume differences are recorded. The density of each SA@yeast bead is measured by using the density calculation formula.
2. N/A means the whole microspheres cannot form.
3. N/D means the experiments were not done.
4. The experiment was carried out by determining the amount of GFP absorbed by 40 SA@yeast beads, when supplying the same amount of GFP. The GFP proteins absorbed by entire 4 were defined as the control.


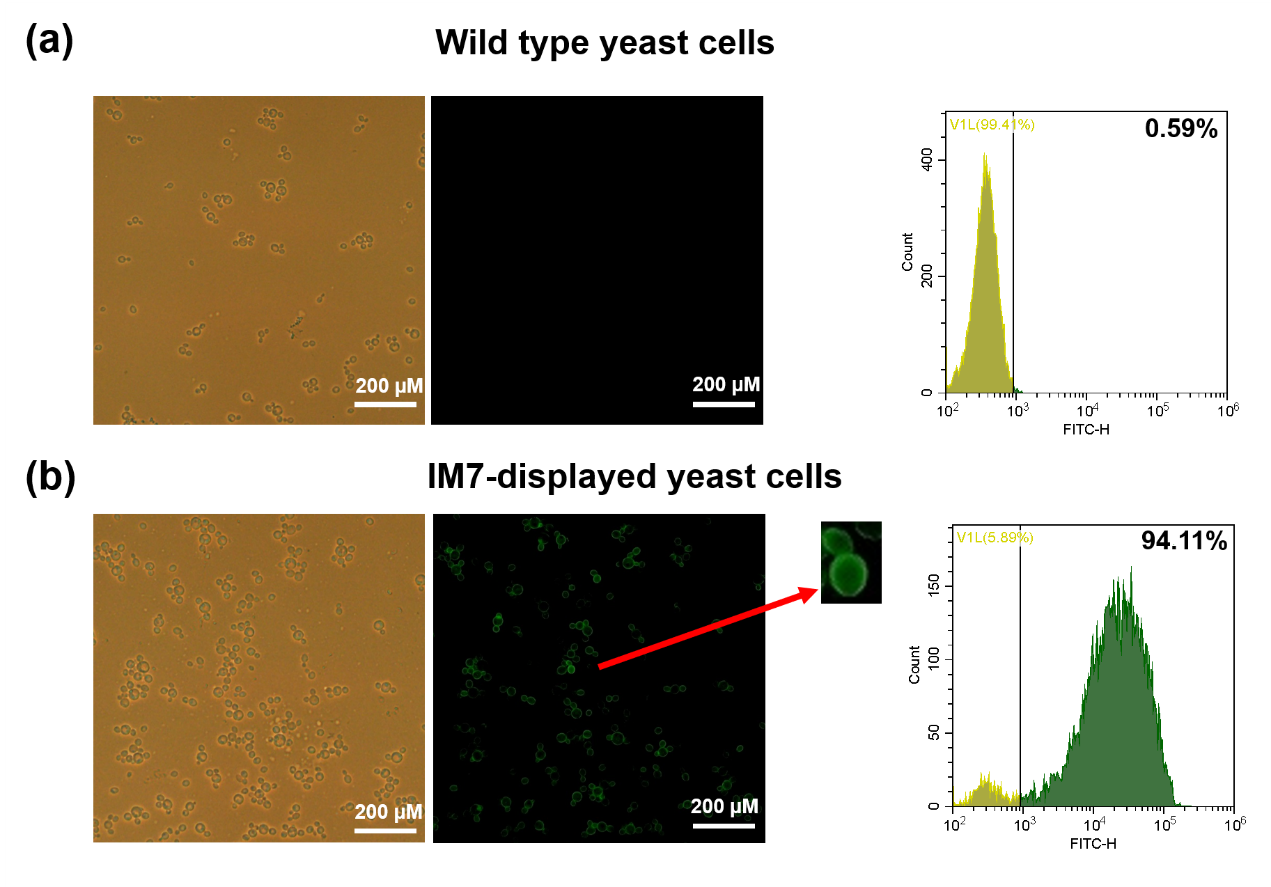


**Figure S1.** Fluorescence microscopy and flow cytometry analysis of IM7-displaying yeast cells. All the yeast cells were treated with mouse anti-HA tag monoclonal antibodies together with FITC (fluorescein isothiocyanate)-conjugated goat anti-mouse IgG antibodies. The inset shows the magnifocation of two fluorescent yeast cells.


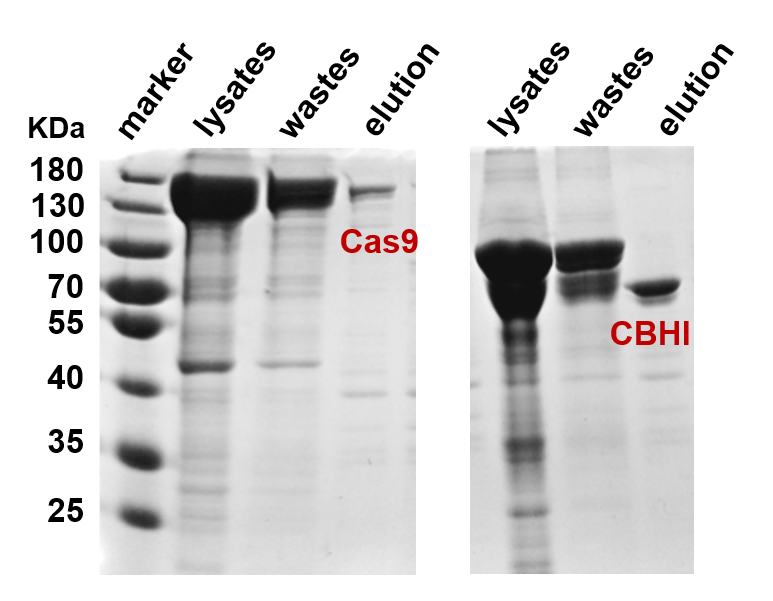


**Figure S2.** 10% SDS-PAGE analysis of Cas9 and CBHI purified by using the Ca-alginate yeast-encapsulated beads.


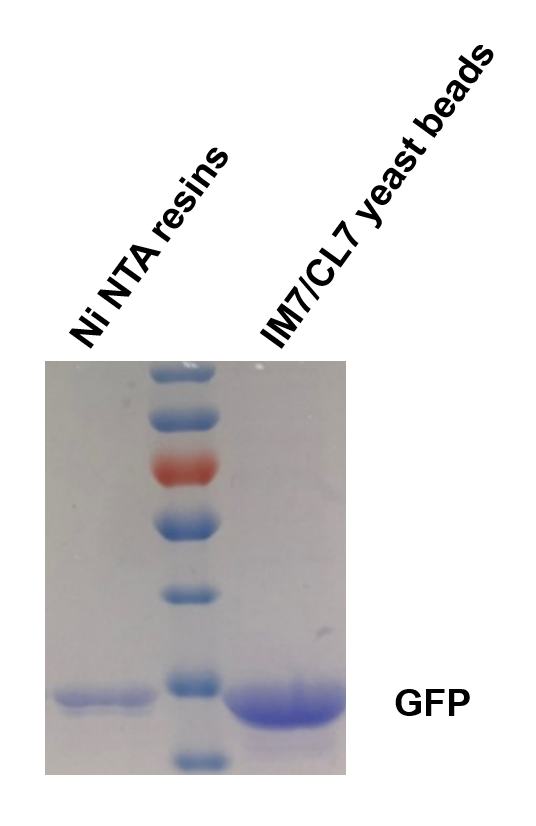


**Figure S3.** 10% SDS-PAGE analysis of GFP purified by using Ni-NTA resin or IM7/CL7 yeast beads.
